# Hypogammaglobulinaemia during rituximab treatment in multiple sclerosis: A Swedish cohort study

**DOI:** 10.1111/ene.16331

**Published:** 2024-05-25

**Authors:** Susanna Hallberg, Björn Evertsson, Ellen Lillvall, Malin Boremalm, Pierre de Flon, Yunzhang Wang, Jonatan Salzer, Jan Lycke, Katharina Fink, Thomas Frisell, Faiez Al Nimer, Anders Svenningsson

**Affiliations:** ^1^ Department of Clinical Sciences Karolinska Institutet, Danderyds Sjukhus Stockholm Sweden; ^2^ Department of Clinical Neuroscience Karolinska Institutet Stockholm Sweden; ^3^ Department of Clinical Neuroscience, Institute of Neuroscience and Physiology at Sahlgrenska Academy University of Gothenburg Gothenburg Sweden; ^4^ Department of Clinical Science, Neurosciences Umeå University Umeå Sweden; ^5^ Department of Clinical Sciences, Neurosciences, Unit of Neurology, Östersund Umeå University Umeå Sweden; ^6^ Clinical Epidemiology Division, Department of Medicine Solna Karolinska Institutet Stockholm Sweden

**Keywords:** disease‐modifying therapy, hypogammaglobulinaemia, IgG decrease, IgM decrease, immunoglobulin decrease, multiple sclerosis, real‐world data, rituximab therapy

## Abstract

**Background and purpose:**

Mechanisms behind hypogammaglobulinaemia during rituximab treatment are poorly understood.

**Methods:**

In this register‐based multi‐centre retrospective cohort study of multiple sclerosis (MS) patients in Sweden, 2745 patients from six participating Swedish MS centres were identified via the Swedish MS registry and included between 14 March 2008 and 25 January 2021. The exposure was treatment with at least one dose of rituximab for MS or clinically isolated syndrome, including data on treatment duration and doses. The degree of yearly decrease in immunoglobulin G (IgG) and immunoglobulin M (IgM) levels was evaluated.

**Results:**

The mean decrease in IgG was 0.27 (95% confidence interval 0.17–0.36) g/L per year on rituximab treatment, slightly less in older patients, and without significant difference between sexes. IgG or IgM below the lower limit of normal (<6.7 or <0.27 g/L) was observed in 8.8% and 8.3% of patients, respectively, as nadir measurements. Six out of 2745 patients (0.2%) developed severe hypogammaglobulinaemia (IgG below 4.0 g/L) during the study period. Time on rituximab and accumulated dose were the main predictors for IgG decrease. Previous treatment with fingolimod and natalizumab, but not teriflunomide, dimethyl fumarate, interferons or glatiramer acetate, were significantly associated with lower baseline IgG levels by 0.80–1.03 g/L, compared with treatment‐naïve patients. Switching from dimethyl fumarate or interferons was associated with an additional IgG decline of 0.14–0.19 g/L per year, compared to untreated.

**Conclusions:**

Accumulated dose and time on rituximab treatment are associated with a modest but significant decline in immunoglobulin levels. Previous MS therapies may influence additional IgG decline.

## INTRODUCTION

B‐cell depleting therapies in autoimmune disease are considered to be well tolerated and efficient [1, 2, 3, 4, 5], but prolonged B‐cell depletion can lead to immunological complications [6, 7]. Treatment‐induced hypogammaglobulinaemia is described in several autoimmune conditions, for example rheumatoid arthritis (RA), neuromyelitis optica spectrum disorders (NMOSD) [8, 9, 10] and multiple sclerosis (MS) [7, 11, 12, 13, 14]. In RA, concomitant therapy is usually administered (e.g., methotrexate) but in MS and NMOSD rituximab is used as a monotherapy. Likewise, increased susceptibility to infections has been described in anti‐CD20 therapies for MS [3, 11, 15, 16, 17, 18], NMOSD [9, 19, 20] and RA [6, 8, 21], linked to decreasing levels of immunoglobulins [6, 17, 22], lymphopenia [18], late‐onset neutropenia [23], reduced vaccine response [24] or coexisting illnesses. Hypogammaglobulinaemia has been suggested to contribute to MS fatigue [25].

Rituximab is a chimeric anti‐CD20 antibody, depleting circulating CD20^+^ B‐lymphocytes [3, 26]. Several randomized and observational studies have established rituximab as a safe and efficient treatment option for MS [1, 3, 4, 27, 28, 29, 30]. Currently used by 59% of all MS patients with an ongoing disease‐modifying therapy (DMT) in Sweden (January 2024, www.neuroreg.se), off‐label use started more than 10 years ago with treatment protocols adapted from rheumatology (1000–2000 mg every 6–24 months). Gradually, lower doses and prolonged intervals were introduced with evidence of sustained efficiency [2, 31, 32, 33].

During pregnancy, immunoglobulin levels decrease physiologically by about 50% from the second trimester, returning to pre‐pregnancy levels after delivery [34].

There is a need for more knowledge regarding the effect of long‐term rituximab treatment on immunoglobulin levels in patients with MS and whether specific patient or treatment‐related factors contribute to the risk of developing decreasing levels of immunoglobulins.

## METHODS

This multi‐centre retrospective cohort study collected data from MS patients in Sweden with rituximab treatment. Patients were eligible for inclusion if diagnosed with MS or clinically isolated syndrome, had received at least one dose of rituximab at a participating centre and were monitored in the Swedish MS Registry (SMSreg).

Three centres in Stockholm (Danderyd Hospital, Karolinska University Hospital, Academic Specialist Centre), one centre in Gothenburg (Sahlgrenska University Hospital) and two centres in Northern Sweden (Umeå University Hospital and Östersund Hospital) participated in this study. Inclusion started from the first registered dose of rituximab, on 14 March 2008, with follow‐up until 25 January 2021. Patients who received plasmapheresis or intravenous immunoglobulin (IVIG) less than 100 days (circa five half‐lives of immunoglobulins) before a rituximab dose were excluded. There were no available data on pre‐existing immunodeficiencies, for example common variable immunodeficiency. The awareness of immunoglobulin measurements was lower during the first study years when pre‐infusion measurements of immunoglobulins did not occur in clinical routine. However, it was possible to collect several immunoglobulin measurements from the diagnostic lumbar puncture occurring before the treatment started. When pre‐treatment assessments of immunoglobulin levels started, immunoglobulin measurements below the normal range were uncommon in the cohort (Figure S1).

### Clinical and immunological variables

Patient characteristics, for example age at first rituximab infusion, sex, disease type (relapsing–remitting, RRMS; secondary progressive, SPMS; and primary progressive, PPMS), disease duration and Expanded Disability Status Scale (EDSS) level at inclusion were collected from SMSreg, with information of doses and dates of previous and current DMTs.

Laboratory results were obtained from patient charts, collecting all available immunoglobulin G (IgG) and immunoglobulin M (IgM) measurements. Laboratory parameters were paired with a subsequent infusion within 45 days.

### Censoring

Patients receiving IVIG infusions for any cause were right censored, contributing with time on treatment and laboratory results until the start of IVIG. Females with a registered pregnancy contributed with time on treatment and laboratory results until conception. Patients transferred to a non‐participating centre were censored at the transfer date. Follow‐up was ended on the day of the last registered rituximab dose. Patients in the Northern, Gothenburg and Stockholm Danderyd cohorts were right censored on 25 January 2021 and in the Stockholm Karolinska/ASC cohort on 14 May 2020.

### Pregnancy

Pregnancy data coverage was not complete in the SMSreg. There were 44 registered pregnancies during the study period. Fertile female patients with transient lowering of IgG levels were therefore additionally assessed through journals for unregistered pregnancies.

### Ethics

Ethics approvals were granted by the Local Ethics Board in Stockholm in 2018 (2018/1286‐31) with amendments by the Swedish Ethical Review Authority in 2019 (2019‐01187).

### Data management and storage

Data were pseudonymized and stored in Excel with a secure login.

### Main outcomes and measurements

The primary outcome was the mean reduction of IgG and IgM levels in relation to time on rituximab treatment. Secondary outcomes were IgG and IgM decrease per 1000 mg of accumulated dose of rituximab and IgG and IgM decrease in relation to the measured covariates baseline IgG value, previous exposure to immunomodulatory drugs, age when starting rituximab treatment, centre and sex. The risk of developing hypogammaglobulinaemia below the lower limit of normal (LLN) (IgG <6.7 g/L) or low IgM (<0.27 g/L), categorized as mild (IgG 4–6.6 g/L) or severe (<4.0 g/L) hypogammaglobulinaemia, was evaluated. The laboratory reference interval at the 2.5th centile for IgG and IgM was used as a cut‐off measurement, reflecting the clinical threshold for detecting patients at risk of imminent immunodeficiency.

### Statistical methods

Linear regression was used to analyse the relationship between the outcomes IgG and IgM levels regarding exposure variables age, sex, centre and previous DMT at baseline.

Associations between exposures time on rituximab (in years) or accumulated dose on rituximab (in grams) and outcome variables (age, sex, centre, previous DMT) were analysed by linear regression at baseline, adjusting for covariates. Mean values were used to describe normally distributed data with standard deviations (SD). Medians and interquartile ranges (IQRs) were used to describe non‐normally distributed values. *p* values under 0.05 were considered significant. Statistical analyses were performed in R 4.2.3.

Generalized estimating equation models were used to examine the relationships between the outcomes IgG and IgM levels and the main exposure time on rituximab (in years) whilst adjusting for additional predictors, including age, sex, previous DMTs and centre. Confidence intervals were corrected for repeated measurements on individuals through robust ‘sandwich’ standard errors, implemented by setting the correlation matrix to ‘independence’. In two separate models for IgG/IgM, the main exposure was changed to the total accumulated dose of rituximab. Additionally, for the IgM models, quadratic terms were included for both the time from the rituximab treatment start and total rituximab dosage to investigate the nonlinear effects (*geeglm* function in R *geepack* package). As a sensitivity analysis, the model was fitted with a truncated dataset for different follow‐up times. Censoring at 3 and 5 years after the first rituximab dose, no significant impact in length of follow‐up time on the estimated mean change over time was found.

Data were checked for outliers (above 1.5 times the upper IQR for IgG and IgM) and apparent mistyped values were corrected (IgG and IgM values mixed up; B‐lymphocyte count data input as 10^6^ instead of 10 [9]). No outliers were excluded.

## RESULTS

In all, 2752 patients were identified at the participating centres in Sweden treated with rituximab between 2008 and 2021. After removing five duplicates (transferring between participating centres) and two patients lost to follow‐up (non‐retrievable data after moving to another centre), the cohort consisted of 2745 patients (2134 women, 611 men): Stockholm *n* = 1869, Gothenburg *n* = 417 and Northern region *n* = 458. In total, 13,155 IgG and 8180 IgM measurements were paired with a subsequent infusion. The mean number of IgG measurements per patient was 5.0 (SD 2.6) and the mean number of IgM measurements was 3.9 (SD 2.3) (Figure S2).

### Baseline characteristics and previous DMT

Demographics at the rituximab treatment start and rituximab exposure variables are summarized in Table 1. The mean age at the first rituximab dose was 42.1 (SD = 11.6) years with 77.7% (*n* = 2134) female. The median EDSS level was 2.5 at inclusion (IQR 1.5–4.0). A majority, 74.3% (*n* = 2040), had RRMS, 20.0% SPMS and 5.1% PPMS. The median disease duration was 8.2 (2.8–16.0) years and the median time from diagnosis to rituximab start was 4.7 (0.5–11.4) years. 32% (*n* = 878) of patients were previously untreated. The most recent prior treatments registered were natalizumab 21.3% (*n* = 585), interferon β 19.6% (*n* = 537), dimethyl fumarate 9.4% (*n* = 258), fingolimod 8.5% (*n* = 234), glatiramer acetate 4.0% (*n* = 109) and teriflunomide 1.8% (*n* = 50). Fewer than 50 patients each were previously treated with an unspecified DMT (*n* = 43), mitoxantrone (*n* = 35), alemtuzumab (*n* = 12) or switched from autologous hematopoietic stem cell transplant, daclizumab, azathioprine or ciclosporin (*n* = 1 each) and were not included in baseline immunoglobulin analyses.

**TABLE 1:** Baseline characteristics and exposure variables for the hypogamma‐MS cohort.

	Stockholm	Northern region	Gothenburg	Overall
	(*N* = 1870)	(*N* = 458)	(*N* = 417)	(*N* = 2745)
Sex				
Female	1550 (82.9%)	310 (67.7%)	274 (65.7%)	2134 (77.7%)
Male	320 (17.1%)	148 (32.3%)	143 (34.3%)	611 (22.3%)
Age				
Mean (SD)	42.0 (11.4)	41.0 (11.5)	43.7 (12.3)	42.1 (11.6)
Median (min, max)	41.9 (14.4, 77.8)	41.6 (13.5, 75.1)	43.0 (9.73, 74.6)	42.1 (9.73, 77.8)
MS subtype				
RRMS	1397 (74.7%)	355 (77.5%)	288 (69.1%)	2040 (74.3%)
SPMS	383 (20.5%)	74 (16.2%)	92 (22.1%)	549 (20.0%)
PPMS	75 (4.0%)	29 (6.3%)	37 (8.9%)	141 (5.1%)
NA	15 (0.8%)	0 (0%)	0 (0%)	15 (0.5%)
EDSS				
Mean (SD)	2.88 (2.06)	2.58 (1.95)	3.70 (2.19)	2.96 (2.09)
Median (min, max)	2.50 (0, 9.00)	2.00 (0, 9.00)	3.00 (1.00, 9.00)	2.50 (0, 9.00)
Missing	219 (11.7%)	55 (12.0%)	39 (9.4%)	313 (11.4%)
Previous DMT				
Treatment‐naïve	577 (30.9%)	178 (38.9%)	123 (29.5%)	878 (32.0%)
Natalizumab	361 (19.3%)	89 (19.4%)	135 (32.4%)	585 (21.3%)
Interferon β	375 (20.1%)	117 (25.5%)	45 (10.8%)	537 (19.6%)
Dimethyl fumarate	211 (11.3%)	8 (1.7%)	39 (9.4%)	258 (9.4%)
Fingolimod	187 (10.0%)	21 (4.6%)	26 (6.2%)	234 (8.5%)
Glatiramer acetate	76 (4.1%)	21 (4.6%)	12 (2.9%)	109 (4.0%)
Other	47 (2.5%)	23 (5.0%)	24 (5.8%)	94 (3.4%)
Teriflunomide	36 (1.9%)	1 (0.2%)	13 (3.1%)	50 (1.8%)
IgG				
Mean (SD)	10.5 (2.28)	10.6 (2.23)	10.2 (2.22)	10.5 (2.27)
Median (min, max)	10.3 (1.50, 20.9)	10.4 (5.70, 18.8)	10.0 (4.40, 16.0)	10.3 (1.50, 20.9)
Missing	314 (16.8%)	296 (64.6%)	230 (55.2%)	840 (30.6%)
IgM				
Mean (SD)	1.10 (0.635)	1.05 (0.595)	1.10 (0.761)	1.10 (0.635)
Median (min, max)	0.97 (0, 5.15)	0.99 (0.14, 3.2)	0.85 (0.26, 3.7)	0.97 (0, 5.15)
Missing	372 (19.9%)	390 (85.2%)	397 (95.2%)	1159 (42.2%)
Time diagnosis to RTX start (years)				
Mean (SD)	7.00 (7.28)	5.50 (6.28)	7.73 (7.22)	6.85 (7.14)
Median (min, max)	4.84 (−1.76, 43.1)	3.43 (−0.397, 31.0)	6.20 (−1.27, 35.1)	4.74 (−1.76, 43.1)
Missing	53 (2.8%)	4 (0.9%)	20 (4.8%)	77 (2.8%)
Time from diagnosis to RTX start (years)				
Mean (SD)	7.00 (7.28)	5.50 (6.28)	7.73 (7.22)	6.85 (7.14)
Median (min, max)	4.84 (−1.76, 43.1)	3.43 (−0.397, 31.0)	6.20 (−1.27, 35.1)	4.74 (−1.76, 43.1)
Missing	53 (2.8%)	4 (0.9%)	20 (4.8%)	77 (2.8%)
Time on RTX treatment				
Mean (SD)	1010 (664)	1700 (865)	1070 (715)	1130 (753)
Median (min, max)	984 (0, 3150)	1690 (0, 4170)	978 (0, 4320)	1100 (0, 4320)
Received doses of RTX				
Mean (SD)	5.90 (3.16)	7.35 (2.76)	6.25 (3.35)	6.19 (3.17)
Median (min, max)	6.00 (1.00, 25.0)	8.00 (1.00, 28.0)	6.00 (1.00, 22.0)	6.00 (1.00, 28.0)
Accumulated dose of RTX				
Mean (SD)	3240 (1870)	5720 (2550)	4430 (2730)	3830 (2340)
Median (min, max)	3000 (500, 12,600)	5900 (500, 15,000)	3500 (1000, 19,500)	3500 (500, 19,500)

Abbreviations: DMT, disease‐modifying therapy; EDSS, Expanded Disability Status Scale; IgG, immunoglobulin G; IgM, immunoglobulin M; MS, multiple sclerosis; NA, Not Available; PPMS, primary progressive multiple sclerosis; RRMS, relapsing–remitting multiple sclerosis; RTX, rituximab; SPMS, secondary progressive multiple sclerosis.

### Treatment regimens

The mean time on rituximab treatment was 3.1 (1.0–5.2) years, and the mean accumulated dose of rituximab was 3830 mg (SD = 2340), displayed in Table 1. The median number of rituximab infusions was 6 (range 1–28) (Figure S3). Patients received 500–1000 mg intravenously every 6–12 months. Higher initial treatment doses were used initially, commencing in the Northern region. The dosing regimen has gradually decreased over time (Figure S4). The median start dose was 1000 g, and the mean start dose was 804 g (SD = 350). The median interval between doses was 183 to 245 days (Table S3).

### Effect on immunoglobulin levels

**FIGURE 1:**
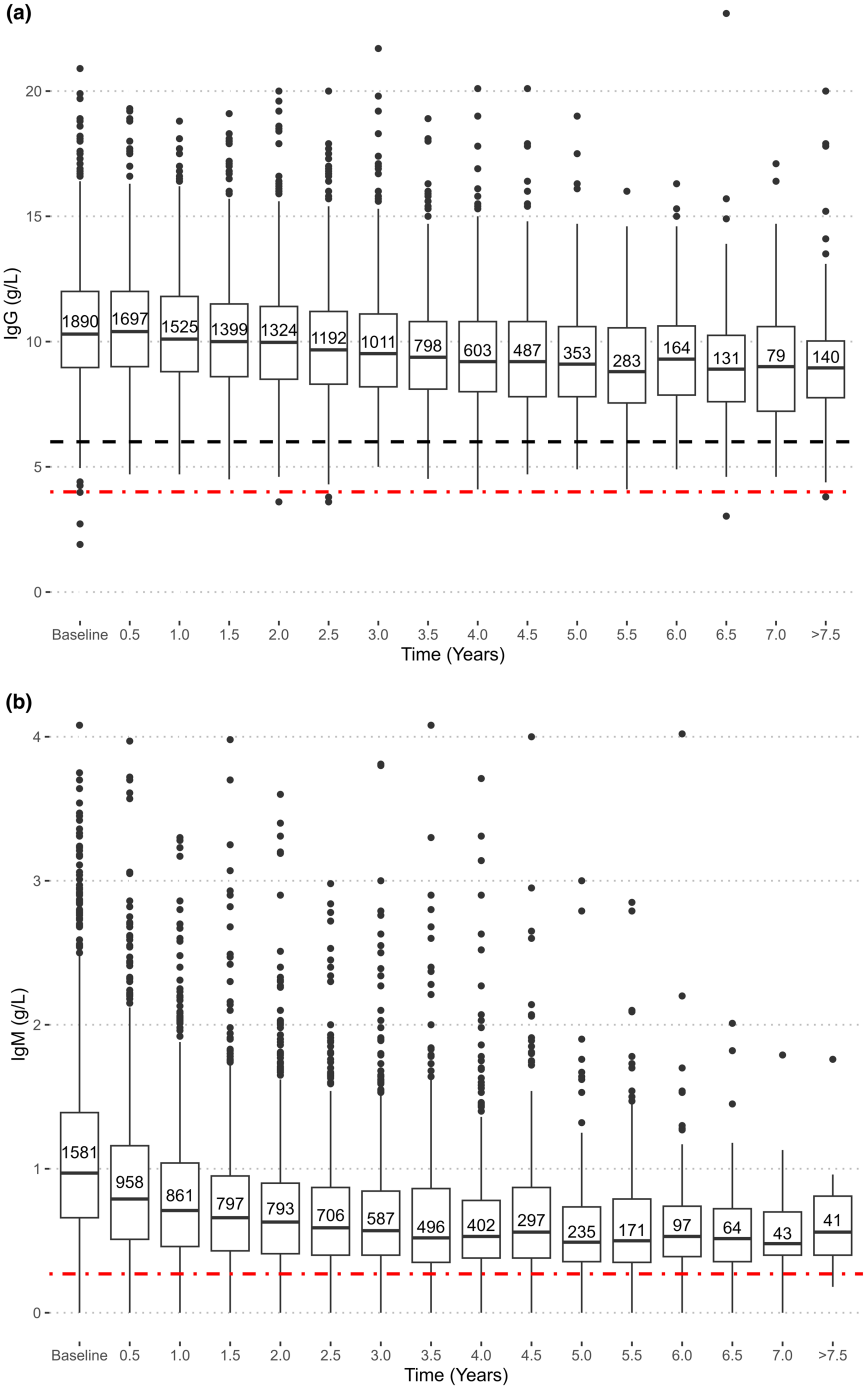
Serum levels of IgG (a) and IgM (b) decline as a function of time on treatment with rituximab. The boxes display the median and interquartile (IQR) range at each time point. Whiskers denote 1.5 × IQR and dots indicate outliers or individual values outside this range. The dashed black line indicates the lower limit of normal (LLN) for IgG at our laboratory (6.7 g/L). The red dash‐dot line in (a) indicates the level of severe hypogammaglobulinaemia (<4 g/L) and in (b) LLN (0.27 g/L).

The mean IgG decrease for the entire cohort was 0.27 (95% confidence interval 0.17–0.36) g/L per year on rituximab therapy (Table 2) when controlling for known factors, for example pregnancy‐induced immunoglobulin decrease, plasmapheresis, IVIG therapy, age and sex. The mean IgG decrease was linear over time (Figure 1a), but IgM was nonlinear (Figure 1b, Table 3). The mean IgG decrease was also analysed in relation to the accumulated dose of rituximab, with a decrease of 0.18 (95% confidence interval 0.16–0.20) g/L per 1000 mg of administered rituximab (Table 4). Inter‐individual differences and IgG decrease patterns are displayed in a spaghetti plot (Figure S1).

**TABLE 2:** Analyses of factors associated with decline of IgG during rituximab treatment assuming time on treatment as an independent variable.

Predictors	Estimates	Confidence interval	*p*
**Intercept (g/L)**	10.88	10.63–11.13	**<0.001**
**Rate of immunoglobulin change in untreated, females and age of lowest quantile, per year (g/L/year)**	−0.27	−0.36 to −0.17	**<0.001**
***Additional change in rate per year (g/L/year)***			
× Sex (Male)^a^	0.07	−0.04 to 0.18	0.240
× Age, per year (2nd quantile)^b^	0.08	−0.01 to 0.16	0.082
× Age, per year (3rd quantile)^b^	0.08	−0.00 to 0.17	0.053
× Age, per year (4th quantile)^b^	0.13	−0.00 to 0.27	0.058
× Previous DMT (Fingolimod)^c^	0.02	−0.10 to 0.15	0.697
× Previous DMT (Natalizumab)^c^	−0.01	−0.13 to 0.12	0.928
× Previous DMT (Teriflunomide)^c^	−0.18	−0.46 to 0.10	0.211
× Previous DMT (Other)^c^	−0.06	−0.30 to 0.18	0.610
× Previous DMT (Dimethyl fumarate)^c^	−0.18	−0.33 to −0.03	**0.016**
× Previous DMT (Glatiramer acetate)^c^	−0.13	−0.28 to 0.03	0.107
× Previous DMT (Interferon β)^c^	−0.14	−0.23 to −0.05	**0.002**
***Effect on baseline***			
**Age, quantiles (g/L)**			
2nd quantile^b^	0.11	−0.17 to 0.38	0.458
3rd quantile^b^	−0.09	−0.39 to 0.20	0.535
4th quantile^b^	−0.65	−0.97 to −0.32	**<0.001**
**Sex (g/L)**			
Male^a^	−0.22	−0.49 to 0.05	0.109
**Previous DMT (g/L)**			
Fingolimod^c^	−1.03	−1.38 to −0.67	**<0.001**
Natalizumab^c^	−0.80	−1.10 to −0.49	**<0.001**
Teriflunomide^c^	−0.41	−1.15 to 0.33	0.276
Other^c^	−0.09	−0.78 to 0.60	0.807
Dimethyl fumarate^c^	−0.03	−0.37 to 0.31	0.868
Glatiramer acetate^c^	0.18	−0.36 to 0.73	0.507
Interferon β^c^	0.40	0.10–0.71	**0.009**
**N**_**ID**_	2630		
**Observations**	13076		

Abbreviations: DMT, disease‐modifying therapy, with treatment‐naïve patients as the reference group; g/L, grams per liter; GEE, generalized estimating equations; IgG, immunoglobulin G; NID, number of Individuals.

*Note*: The reference group for sex is females, and for age, it is the lowest quantile. The statistical model employed, Generalized Estimating Equations (GEE), was chosen due to its robustness in handling the correlated data typical of longitudinal studies, assuming linear relationships among variables. A significance level of *p* < 0.05 was adopted, but given the 23 comparisons made, a Bonferroni‐adjusted threshold of 0.002 (0.05/23) could be used to mitigate the risk of Type I error. This adjustment necessitates a cautious interpretation of findings near this significant boundary.

^a^ The comparison group is female pwMS.

^b^ The comparison group is the lowest quantile of age at inclusion.

^c^ The comparison group is previously untreated pwMS.

**TABLE 3:** Analyses of factors associated with decline of IgM during rituximab treatment assuming time on treatment as an independent variable.

Predictors	Estimates	Confidence interval	*p*
**Intercept (g/L)**	1.23	1.16–1.31	**<0.001**
**Rate of immunoglobulin change in untreated, females and age of lowest quantile, per year (g/L/year)**	−0.24	−0.27 to 0.20	**<0.001**
Squared “Rate of immunoglobulin change in untreated, females and age of lowest quantile”	0.03	0.02–0.03	**<0.001**
***Additional change in rate per year (g/L/year)***			
× Sex (Male)^a^	−0.02	−0.05 to −0.00	**0.031**
× Age (2nd quantile)^b^	0.03	0.00–0.05	**0.043**
× Age (3rd quantile)^b^	0.03	0.00–0.06	**0.043**
× Age (4th quantile)^b^	0.06	0.03–0.08	**<0.001**
× Previous DMT (Natalizumab)^c^	0.03	−0.00 to 0.06	0.078
× Previous DMT (Fingolimod)^c^	0.03	0.00–0.06	**0.047**
× Previous DMT (Teriflunomide)^c^	0.00	−0.07 to 0.06	0.898
× Previous DMT (Other)^c^	0.00	−0.06 to 0.06	0.889
× Previous DMT (Dimethyl fumarate)^c^	0.01	−0.03 to 0.05	0.509
× Previous DMT (Glatiramer acetate)^c^	0.01	−0.03 to 0.04	0.762
× Previous DMT (Interferon β)^c^	0.00	−0.03 to 0.04	0.906
***Effect on baseline***			
**Age, quantiles (g/L)**			
2nd quantile^b^	−0.08	−0.16 to 0.00	0.065
3rd quantile^b^	−0.04	−0.14 to 0.05	0.381
4th quantile^b^	−0.2	−0.29 to −0.11	**<0.001**
**Sex (g/L)**			
Male^a^	0.02	−0.05 to 0.09	0.615
**Previous DMT (g/L)**			
Natalizumab^c^	−0.31	−0.40 to −0.23	**<0.001**
Fingolimod^c^	−0.30	−0.40 to −0.21	**<0.001**
Teriflunomide^c^	−0.11	−0.29 to 0.07	0.234
Other^c^	−0.2	−0.39 to −0.01	**0.035**
Dimethyl fumarate^c^	−0.02	−0.13 to 0.08	0.662
Glatiramer acetate^c^	−0.11	−0.23 to 0.02	0.094
Interferon β^c^	−0.06	−0.16 to 0.03	0.191
**N**_**ID**_	2072		
**Observations**	8129		

*Note*: Since the decline of IgM is not linear a square term “Rate of immunoglobulin change in untreated, females and age of lowest quantile” to adjust for this non‐linearity. The Bonferroni‐adjusted threshold of 0.002 (0.05/24) because of the added squared term.

^a^ The comparison group is female pwMS.

^b^ The comparison group is the lowest quantile of age at inclusion.

^c^ The comparison group is previously untreated pwMS.

**TABLE 4:** Analyses of factors associated with decline of IgG during rituximab treatment assuming accumulated dose of rituximab as independent variable.

Predictors	Estimates	Confidence interval	*p*
**Intercept (g/L)**	10.91	10.66–11.15	**<0.001**
**Rate of immunoglobulin change in untreated females and age of lowest quantile, per 1000 mg accumulated dose rituximab (g/L/1000 mg)**	−0.19	−0.23 to −0.16	**<0.001**
***Effect on baseline***			
**Age, quantiles (g/L)**			
2nd quantile^b^	0.27	0.03–0.51	**0.029**
3rd quantile^b^	0.09	−0.16 to 0.33	0.501
4th quantile^b^	−0.40	−0.67 to −0.13	**0.003**
**Sex (g/L)**			
Male^a^	−0.03	−0.25 to 0.18	0.763
**Previous DMT (g/L)**			
Fingolimod^c^	−0.98	−1.29 to −0.67	**<0.001**
Natalizumab^c^	−0.56	−0.81 to −0.30	**<0.001**
Teriflunomide^c^	−0.71	−1.37 to −0.05	**0.035**
Dimethyl fumarate^c^	−0.32	−0.66 to 0.01	0.059
Glatiramer acetate^c^	−0.10	−0.55 to 0.35	0.651
Other^c^	−0.25	−0.79 to 0.29	0.368
Interferon β^c^	0.07	−0.19 to 0.32	0.606
**N**_**ID**_	2630		
**Observations**	13076		

Abbreviations: BEAM, Bi‐ethylhexylamine; CI, Confidence interval; DMT, Disease‐Modifying Therapy; g/L, grams per liter; HSCT, Hematopoietic Stem Cell Transplantation; IgG, Immunoglobulin G; NID, Number of Individuals.

*Note*: Estimates were calculated using a Generalized Estimating Equations (GEE) model to accommodate repeated measures and within‐subject correlation. The 'Rate of immunoglobulin change' is expressed per 1000 mg of accumulated rituximab dose. The category 'Other treatments' under previous DMT includes therapies such as Azathioprine, HSCT/BEAM, Cyclosporine, Daclizumab, clinical trial medications, Mitoxantrone, and Alemtuzumab. These are consolidated due to smaller patient numbers for each treatment and are referred to as 'Other' in the dataset. Age and sex effects on baseline IgG are presented as changes in g/L. *p*‐Values <0.05 are considered statistically significant and indicate the likelihood that the observed effects are not due to random chance. The analysis includes 2630 individuals contributing to a total of 13076 observations, allowing for a comprehensive evaluation of the impact of rituximab dosage, demographic variables, and treatment history on IgG levels.

^a^ The comparison group is female pwMS.

^b^ The comparison group is the lowest quantile of age at inclusion.

^c^ The comparison group is previously untreated pwMS.

**FIGURE 2:**
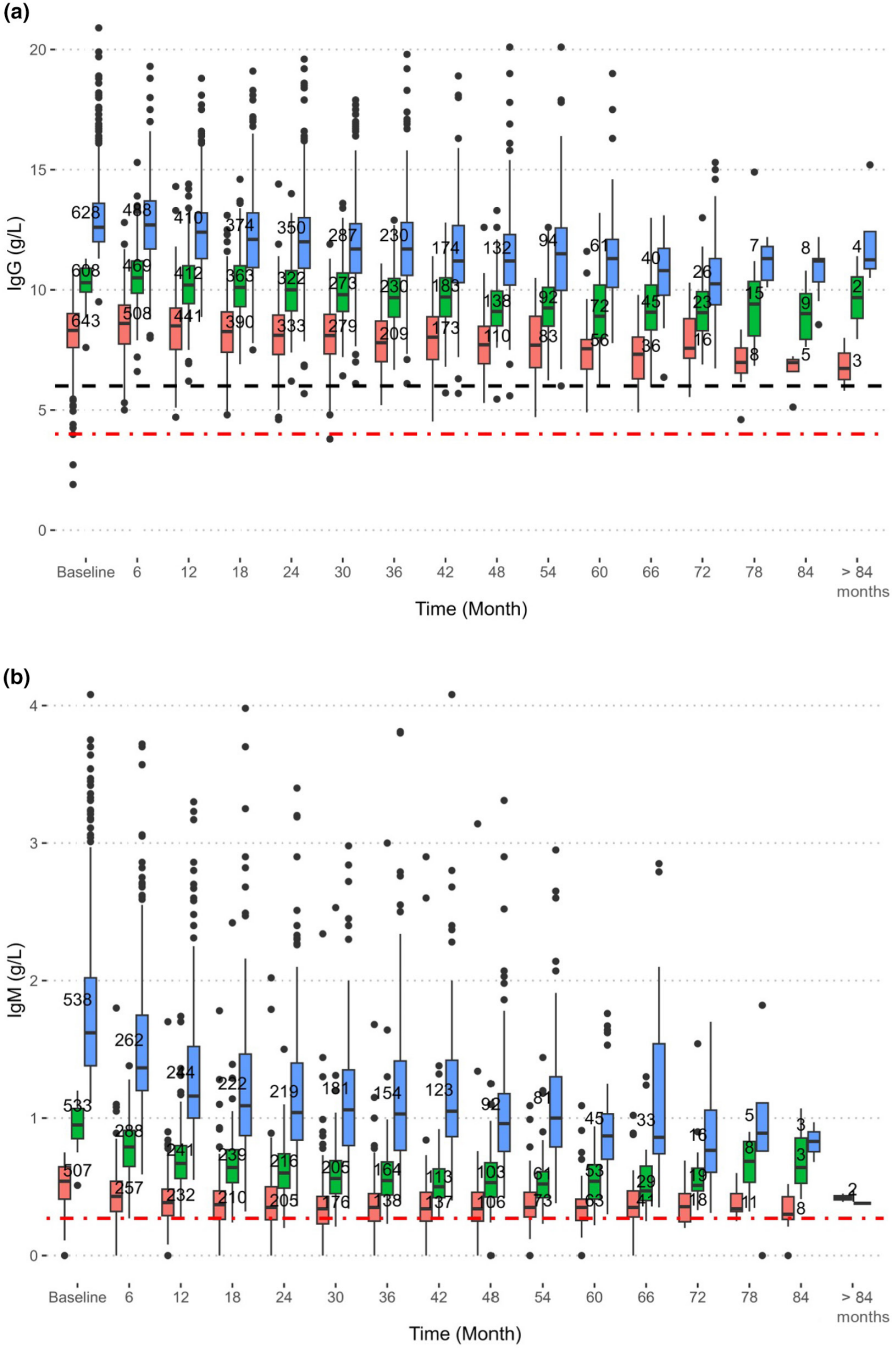
Individuals with higher baseline IgG (a) or IgM (b) tended rather to decline faster in IgG upon a similar rituximab challenge over time compared to individuals with lower baseline values. The boxes display the median and interquartile range (IQR) at each time point. Whiskers denote 1 . 5 × IQR and dots indicate outliers. The dashed black line indicates the lower limit of normal (LLN) for IgG at our laboratory (6.7 g/L). The red dash‐dot line in (a) indicates the level of severe hypogammaglobulinaemia (<4 g/L) and in (b) LLN (0.27 g/L).

There was a small, but significant, decrease of baseline IgG with age at inclusion (Figure 2a), and similar but nonlinear for IgM (Figure 2b). The rate of IgG decrease diminished slightly over time by 0.005 g/L per year of age (data not shown) which could be due to lower dosage protocols for older patients or patient selection. There were no statistically significant differences in IgG measurements between sexes at baseline (Tables 2 and 3).

Analysing immunoglobulin kinetics, those with the lowest baseline IgG and IgM measurements decreased slightly less than those in higher baseline groups (Figure 2a,b; Table S1A,B).

Checking the validity of the models over time, those treated with rituximab for a shorter or longer time did not differ at baseline IgG levels. During follow‐up, there was no difference in IgG decrease between those with a baseline IgG measurement and those without (data not shown). Analysing the interaction between time on treatment and sex, no significant difference between time on rituximab treatment and sex was noted.

Hypogammaglobulinaemia occurred in 8.8% of patients, with a nadir value of IgG below LLN (<6.7 g/L), and six out of 2745 (0.2%) developed severe hypogammaglobulinaemia (IgG <4.0 g/L). Low IgM levels were experienced by 8.3%, with a nadir value below LLN (<0.27 g/L). In patients on rituximab therapy for 3 and 5 years, 7.7% and 9.5% had nadir measurements of IgG below LLN, respectively. Sixteen patients received IVIG infusions. Eleven individuals received repeated IVIG therapy for hypogammaglobulinaemia or infections and five patients received a single treatment and then continued rituximab infusions. There was a trend for previously natalizumab‐ and fingolimod‐treated patients to reach IgG measurements below LLN earlier. No statistically significant difference in time to IgG levels below LLN or severe hypogammaglobulinaemia between sexes was seen.

### Previous treatment and risk of low baseline IgG levels

The impact of previous DMTs for MS on baseline IgG levels before commencing rituximab treatment compared to treatment‐naïve patients was adjusted for age. For those switching from fingolimod, the mean baseline IgG was 1.03 g/L lower, for natalizumab 0.80 g/L lower (Table 2) and for interferon‐β‐treated patients 0.40 g/L higher than for the treatment‐naïve patients. For teriflunomide, glatiramer acetate or dimethyl fumarate, there was no significant difference. For patients previously treated with dimethyl fumarate, the IgG decrease rate was 0.18 g/L/year higher, compared to untreated patients. Similarly, patients treated with interferon β experienced an additional decrease in IgG levels, of 0.14 g/L/year compared to untreated patients.

## DISCUSSION

In this large MS cohort compiling retrospective data from about half of all rituximab‐treated patients in Sweden, mean IgG levels decreased linearly by 0.27 g/L per treatment year. This decrease is interpreted as relatively modest, but its linear nature suggests that it may not be a direct consequence of rituximab treatment. Instead, it could be attributed to the attrition of plasma cells, which are not adequately repopulated by novel B‐cells [35, 36], or by a secondary or downstream effect. Identifying the factors that expedite this plasma cell attrition could provide critical insights for mitigating the risk of diminished IgG levels in patients treated with rituximab. Even with treatment interruption, immunoglobulins remained low or even continued to decrease, and from our clinical experience it may take years for IgG to begin increasing.

There were no differences in immunoglobulin decrease between sexes, despite known differences in drug pharmacokinetics [37]. Furthermore, a small but significant association between age and lower IgG levels at baseline was detected, driven by the patients over 50 years, contrasting with healthy individuals, where older individuals exhibit higher levels of IgG [38].

The IgG decrease during rituximab therapy appeared linear in our population. This agrees with other studies in which starting with a lower value increased the risk of reaching LLN [36, 39]. Surprisingly, individuals with higher baseline IgG and IgM values tended to experience a greater rate of reduction of IgG and IgM over time in our study. There were no significant differences in follow‐up dropout rates in relation to baseline IgG level.

In the present study, natalizumab and fingolimod were identified as predictors for reduced baseline IgG levels in patients with MS. However, it is worth noting that the mechanisms underlying these reductions appear to vary between treatments. Natalizumab has no known effect on B‐cell function besides migration properties. However, other MS studies have reported immunoglobulin decrease during treatment with natalizumab for MS [11, 40, 41, 42]. Possible mechanisms presented have been a natalizumab‐induced alteration of B‐cell gene expression, blocking of VLA4‐mediated interaction of plasma cells in the bone marrow [43], epigenetic changes [42] or interference with B‐cell homing to peripheral lymphoid tissue [41]. Also, significantly lower mean IgG and IgM are noted for fingolimod compared to healthy individuals [11]. Despite these findings, current guidelines do not recommend routine monitoring of IgG levels during treatment with natalizumab and fingolimod. We argue that monitoring immunoglobulin levels in MS therapies may be extended beyond anti‐CD20 therapies.

Hypogammaglobulinaemia secondary to immunosuppressive therapies is increasingly reported in MS and other autoimmune diseases [7, 44]. Repeated dosing of anti‐CD20 therapies not allowing for B‐cell repopulation has been associated with a more rapid decrease of immunoglobulin levels than individual doses. Several recent MS and NMOSD studies report a correlation between time on treatment, treatment dose and the risk of developing hypogammaglobulinaemia [6, 9, 19, 20, 39]. IgG decrease rates of approximately 0.4 g/L yearly are described with induction protocols (2000 mg of rituximab) and re‐treatment immediately after B‐cell repopulation [9, 19, 20]. In a 14‐year NMOSD follow‐up study by Kim et al. [19], 41% experienced IgG measurements below <6.0 g/L (assigned LLN). A majority (11/15) of participants developed IgG levels below <7.0 (assigned LLN) during a 70‐month median follow‐up in an Italian NMOSD cohort [9]. With similar rituximab treatment for up to 3.7 years for myelin oligodendrocyte glycoprotein antibody disorder or NMOSD, 17% of patients experienced hypogammaglobulinaemia (LLN <6.0 g/L), with a median of 5.4 years to hypogammaglobulinaemia onset [20]. In RA, hypogammaglobulinaemia and risk of infections are recognized complications of long‐term B‐cell depleting therapies and are reported in up to 24% of patients [6, 8, 39].

The data from our cohort indicate a lower rate of IgG decline compared with the NMOSD and RA studies, which possibly in part could be explained by dose reductions and extended infusion intervals at Swedish centres [2, 31, 32]. In the rituximab studies for RA, concomitant medication with methotrexate and oral steroids can contribute to this risk [8]. Higher accumulated doses of rituximab and not allowing B‐cell repopulation may account for a higher risk in NMOSD treatment [11, 16, 41, 45]. In NMOSD, there were no available data on other concomitant medications, for example steroids or other immunosuppressants, possibly contributing to IgG decrease [7].

It is essential to manage the increased infection risk in hypogammaglobulinaemia during rituximab therapy [7, 16, 44]. The Swedish real‐world prospective cohort by Luna et al. [17] reported an increased risk of infections requiring hospitalization or antibiotics or antivirals, compared to healthy controls or MS patients treated with injectables, natalizumab and fingolimod. Importantly, these data do not take rituximab treatment length or accumulated dose into account nor analyse the relation with IgG or B‐lymphocyte values. In a French observational study [16] assessing the risk of hypogammaglobulinaemia and infections in 188 MS patients with rituximab treatment, IgG levels <6 g/L were significantly associated with a risk of developing infections. Compared to our study, a higher proportion developed IgG <6 g/L (23.4%) and <4 g/L (4.2%). The number of doses was comparable; however, the median EDSS was higher than in our cohort [16].

Ocrelizumab and ofatumumab [26] were not associated with hypogammaglobulinaemia in the MS registration studies OPERA [46] and ASCLEPIOS [47]. The OPERA ocrelizumab 5‐year extension [5] observed trends towards lower IgG and IgM levels, reporting IgG below assigned LLN (<5.45 g/L) in 5.4% and IgM below assigned LLN (<0.4 g/L) in 29.5%. Most patients received steroids before infusions [47]. In the 3.5‐year extension ofatumumab ALITHIOS [48] study, 1.7% developed IgG below the same LLN (<5.65 g/L) and 25.1% IgM (<0.40 g/L) [48]. The IgG levels decreased by 9.5% at year 1 and 17% at a 5‐year follow‐up of ocrelizumab compared with baseline values, and for ofatumumab IgG levels remained relatively stable [45]. A study comparing rituximab and ocrelizumab during the first year found lower IgG levels in the ocrelizumab group compared to the rituximab group [30]. Notably, LLN was >1 g/L lower in the ocrelizumab and ofatumumab trials than in our study [46, 47, 48].

The presented lower risk for IgG decrease for ofatumumab in the ALITHIOS [48] and ASCLEPIOS [47] studies compared with our data may be an effect of a lower anti‐CD20 antibody dosage. However, it may in part also be explained by differences in follow‐up time and patient selection. Moreover, steroids are not used when administering ofatumumab, which could influence the risk of reducing IgG values [47].

### Strengths and weaknesses

A strength of this real‐world retrospective study is the large number of patients included. Including virtually all patients from three major geographical areas of Sweden treated with rituximab increased the external validity. Data quality was improved by high data density, few switches to other therapies and infusions within a 6‐ or 12‐month timeframe, although the different prevailing infusion schemes over time remain a weakness. Future studies can evaluate whether de‐escalation strategies reduce the risk of developing hypogammaglobulinaemia. In this real‐world setting, there may be different reasons for treatment discontinuation. Only a few patients were followed up on treatment for more than 5 years. Selection bias and healthy survivor bias are some potential confounders in this observational study setting.

Patients remaining on therapy may have been selected for not developing side effects, for example frequent infections. No differences in baseline IgG measurements were found between the whole cohort and those continuing therapy. This strengthens our result of a uniform moderate and gradual decrease in mean IgG levels.

## CONCLUSION

Assessment of possible hypogammaglobulinaemia is important when treating MS. The rate of diminishing immunoglobulins in a large contemporary Swedish cohort during rituximab treatment is described, finding an association with time on treatment and with the total dose of rituximab, with a linear trend towards lower means of IgG levels. At the same time, the IgM decrease is more rapid and nonlinear. The mean IgG levels decrease by 0.27 g/L per year on rituximab therapy. After treatment with rituximab therapy for 3 years and 5 years, 7.7% and 9.5% have a measurement of IgG below LLN (<6.7 g/L), respectively. Previous treatments with natalizumab and fingolimod are associated with lower mean baseline IgG values, which may predict the risk of developing secondary hypogammaglobulinaemia. Switching from interferons and dimethyl fumarate was associated with a more rapid IgG decline, at present of unknown reason and clinical significance.

Future studies need to establish a dosing schedule balancing the risks of negative immunological consequences and treatment efficiency, establishing the optimal B‐cell repopulation before redosing and making pre‐treatment assessments to establish which patients are at risk of secondary hypogammaglobulinaemia.

In an ongoing study, the infection risk in an extended version of this cohort will be analysed in relation to immunoglobulin dynamics, B‐cell subset repopulation patterns and late‐onset neutropenia.

## AUTHOR CONTRIBUTIONS

**Susanna Hallberg:** Conceptualization; investigation; writing – original draft; methodology; validation; visualization; writing – review and editing; software; formal analysis; project administration; data curation; funding acquisition. **Björn Evertsson:** Conceptualization; investigation; funding acquisition; methodology; validation; visualization; writing – review and editing; software; formal analysis; data curation. **Ellen Lillvall:** Validation; writing – review and editing; investigation. **Malin Boremalm:** Investigation; validation; writing – review and editing. **Pierre de Flon:** Investigation; writing – review and editing; validation; data curation. **Yunzhang Wang:** Writing – review and editing; visualization; methodology; software; formal analysis; data curation. **Jonatan Salzer:** Funding acquisition; writing – review and editing; validation; supervision; resources; investigation; methodology. **Jan Lycke:** Funding acquisition; investigation; methodology; validation; writing – review and editing; supervision; resources. **Katharina Fink:** Conceptualization; methodology; writing – review and editing; formal analysis; supervision. **Thomas Frisell:** Methodology; validation; writing – review and editing; formal analysis. **Faiez Al Nimer:** Conceptualization; funding acquisition; methodology; validation; visualization; writing – review and editing; formal analysis; supervision; resources. **Anders Svenningsson:** Conceptualization; investigation; funding acquisition; writing – original draft; methodology; validation; visualization; writing – review and editing; formal analysis; project administration; data curation; supervision; resources.

## FUNDING INFORMATION

The study was financed by grants from the Swedish state under the agreement between the Swedish government and the county councils, the ALF agreement B5069 and by the Neuro Sweden Research Fund. BE was supported by Region Stockholm (combined residency and PhD training programme).

## CONFLICT OF INTEREST STATEMENT

SH, BE, EL, MB, PF, YW, JS, FN and AS reported no conflicts of interest. KF has received lecture honoraria and has served on scientific advisory boards for Biogen, Celgene, Janssen, Merck, Novartis and Roche. JL has received travel support and/or lecture honoraria and has served on scientific advisory boards for Alexion, Almirall, Biogen, Bristol Myers Squibb, Celgene, Janssen, Merck, Novartis, Roche and Sanofi; and has received unconditional research grants from Biogen and Novartis, and financial support from Sanofi for an investigator‐initiated study. TF has received funding from the ARTIS project, a national Swedish safety monitoring of rheumatology immunomodulators, in turn supported by agreements between Karolinska Institutet and Abbvie, BMS, Eli Lilly, Galapagos, MSD, Pfizer, Roche, Samsung Bioepis and Sanofi.

## Supporting information


**Data S1.** Supporting information.

**Figure S1.** A spaghetti plot for the whole cohort displaying the trajectories of all serum IgG values for each participating individual. The figure shows that there is a tendency for declining IgG values over time and that very few individuals display very low values. Red dashed line, LLN (<6.7 g/L); black dashed line, level of severe hypogammaglobulinaemia (<4.0 g/L).
**Figure S2.** Distribution of the number of IgG and IgM values per patient (A) and distribution of the number of rituximab infusions per patient (B). As can be seen in the figure, most individuals contributed with 5–10 immunoglobulin measurements during the study (A). The (B) panel shows that more than half of the cohort received more than six infusions, and the highest number of infusions given to an individual was 28.
**Figure S3.** The four most prevalent dose regimens whilst initiating rituximab treatment in Sweden, with approximate periods included. The timeline displays months since the first rituximab dose. Current guidelines from the Swedish MS Association recommend 1000 mg initially followed by 500 mg 6 monthly for 2 years; after that according to individual assessment.
**Figure S4.** A scatterplot displaying individual baseline IgG measurements (*y*‐axis) in relation to age (*x*‐axis) for treatment‐naïve patients before the start of rituximab therapy. The baseline measurements for all previously untreated individuals with an available IgG measurement are presented as black dots, and the change in mean baseline IgG measurement in relation to age is marked with a blue line. No adjustments of potential cofounders have been performed.
**Table S1A.** IgG decline is dependent on baseline IgG levels before rituximab start. IgG – Immunoglobulin G, g/L – grams per liter, NID – Number of Individuals, Δ – Change in. Baseline IgG levels were categorized into tertials for this analysis, with the lowest tertile serving as the reference category. The Δ IgG represents the average difference in baseline IgG levels for the second and third tertiles compared to the lowest tertile. The rate of immunoglobulin change is reported as an annual decline, calculated from longitudinal data using Generalized Estimating Equations to account for within‐subject correlation over time. The additional rate of IgG change compared with the lowest tertile indicates the difference in the rate of decline between the tertiles. Estimates and 95% confidence intervals reflect the magnitude and precision of the IgG change, with *p*‐values indicating the level of statistical significance for each predictor. A *p*‐value of <0.05 was considered statistically significant. These analyses help to elucidate the dependency of IgG decline on baseline IgG levels prior to rituximab initiation.
**Table S1B.** IgM decline is dependent on baseline IgM levels before rituximab start. This table builds upon the analyses presented in Supplemental Table 1A, extending the investigation to IgM levels. Baseline IgM is similarly divided into tertiles, and the estimated rates of decline are calculated per annum. The lowest tertile serves as the reference for comparison, and the Δ symbol denotes the mean difference in baseline IgM levels for the middle and upper tertiles relative to the lowest. A squared term is included to adjust for non‐linearity in the rate of decline across tertiles.
**Table S2.** Linear regression analysis of age, sex, and prior DMT use on baseline IgG levels in a cohort of before start of rituximab treatment in MS patients.
**Table S3.** Mean and median doses of rituximab (in 1000 milligrams) and intervals between doses (in days) for the consecutive doses 1–13. Number of patients receiving each dose is presented as numbers treated. The lowest and highest received individual dose and interval is presented in brackets.

## Data Availability

The data that support the findings of this study are available from the corresponding author upon reasonable request.
