# Supplementary material for: Hypogammaglobulinaemia during rituximab treatment in multiple sclerosis: A Swedish cohort study
Source: Eur J Neurol. 2024 May 25;31(8):e16331. doi: 10.1111/ene.16331 (PMC11236063; doi:10.1111/ene.16331)
Supplement: Supplementary file 2 — Figure S1. A spaghetti plot for the whole cohort displaying the trajectories of all serum IgG values for each participating individual. The figure shows that there is a tendency for declining IgG values over time and that very few individuals display very low values. Red dashed line, LLN (<6.7 g/L); black dashed line, level of severe hypogammaglobulinaemia (<4.0 g/L). Figure S2. Distribution of the number of IgG and IgM values per patient (A) and distribution of the number of rituximab infusions per patient (B). As can be seen in the figure, most individuals contributed with 5–10 immunoglobulin measurements during the study (A). The (B) panel shows that more than half of the cohort received more than six infusions, and the highest number of infusions given to an individual was 28. Figure S3. The four most prevalent dose regimens whilst initiating rituximab treatment in Sweden, with approximate periods included. The timeline displays months since the first rituximab dose. Current guidelines from the Swedish MS Association recommend 1000 mg initially followed by 500 mg 6 monthly for 2 years; after that according to individual assessment. Figure S4. A scatterplot displaying individual baseline IgG measurements (y‐axis) in relation to age (x‐axis) for treatment‐naïve patients before the start of rituximab therapy. The baseline measurements for all previously untreated individuals with an available IgG measurement are presented as black dots, and the change in mean baseline IgG measurement in relation to age is marked with a blue line. No adjustments of potential cofounders have been performed. Table S1A. IgG decline is dependent on baseline IgG levels before rituximab start. IgG – Immunoglobulin G, g/L – grams per liter, NID – Number of Individuals, Δ – Change in. Baseline IgG levels were categorized into tertials for this analysis, with the lowest tertile serving as the reference category. The Δ IgG represents the average difference in baseline IgG levels for the secon [file ENE-31-e16331-s002.docx]

**Supporting information**

**Data S1.** Supporting information.

**Figure S1.** A spaghetti plot for the whole cohort displaying the trajectories of all serum IgG values for each participating individual. The figure shows that there is a tendency for declining IgG values over time and that very few individuals display very low values. Red dashed line, LLN (<6.7 g/L); black dashed line, level of severe hypogammaglobulinaemia (<4.0 g/L).

**Figure S2.** Distribution of the number of IgG and IgM values per patient (A) and distribution of the number of rituximab infusions per patient (B). As can be seen in the figure, most individuals contributed with 5–10 immunoglobulin measurements during the study (A). The (B) panel shows that more than half of the cohort received more than six infusions, and the highest number of infusions given to an individual was 28.

**Figure S3.** The four most prevalent dose regimens whilst initiating rituximab treatment in Sweden, with approximate periods included. The timeline displays months since the first rituximab dose. Current guidelines from the Swedish MS Association recommend 1000 mg initially followed by 500 mg 6 monthly for 2 years; after that according to individual assessment.

**Figure S4.** A scatterplot displaying individual baseline IgG measurements (*y*‐axis) in relation to age (*x*‐axis) for treatment‐naïve patients before the start of rituximab therapy. The baseline measurements for all previously untreated individuals with an available IgG measurement are presented as black dots, and the change in mean baseline IgG measurement in relation to age is marked with a blue line. No adjustments of potential cofounders have been performed.

**Table S1A.** IgG decline is dependent on baseline IgG levels before rituximab start. IgG – Immunoglobulin G, g/L – grams per liter, NID – Number of Individuals, Δ – Change in. Baseline IgG levels were categorized into tertials for this analysis, with the lowest tertile serving as the reference category. The Δ IgG represents the average difference in baseline IgG levels for the second and third tertiles compared to the lowest tertile. The rate of immunoglobulin change is reported as an annual decline, calculated from longitudinal data using Generalized Estimating Equations to account for within‐subject correlation over time. The additional rate of IgG change compared with the lowest tertile indicates the difference in the rate of decline between the tertiles. Estimates and 95% confidence intervals reflect the magnitude and precision of the IgG change, with *p*‐values indicating the level of statistical significance for each predictor. A *p*‐value of <0.05 was considered statistically significant. These analyses help to elucidate the dependency of IgG decline on baseline IgG levels prior to rituximab initiation.

**Table S1B.** IgM decline is dependent on baseline IgM levels before rituximab start. This table builds upon the analyses presented in Supplemental Table 1A, extending the investigation to IgM levels. Baseline IgM is similarly divided into tertiles, and the estimated rates of decline are calculated per annum. The lowest tertile serves as the reference for comparison, and the Δ symbol denotes the mean difference in baseline IgM levels for the middle and upper tertiles relative to the lowest. A squared term is included to adjust for non‐linearity in the rate of decline across tertiles.

**Table S2.** Linear regression analysis of age, sex, and prior DMT use on baseline IgG levels in a cohort of before start of rituximab treatment in MS patients.

**Table S3.** Mean and median doses of rituximab (in 1000 milligrams) and intervals between doses (in days) for the consecutive doses 1–13. Number of patients receiving each dose is presented as numbers treated. The lowest and highest received individual dose and interval is presented in brackets.

Supplemental Figure S1


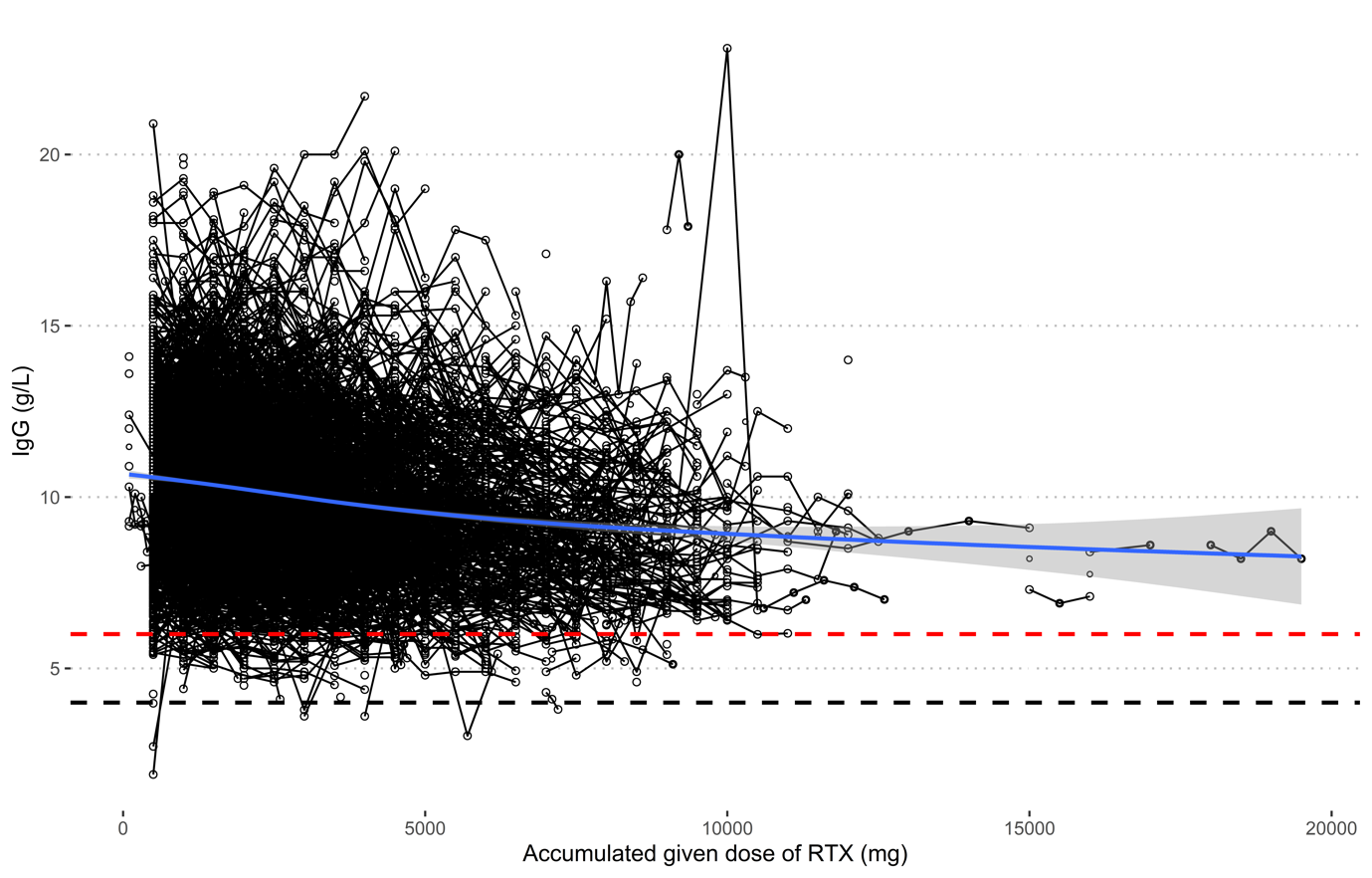


Supplemental Figure S2A

Supplemental Figure S2B

Supplemental Figure S3

Supplemental Figure S4

Supplemental Table S1A

| **Supplemental Table 1A. IgG decline is dependent on baseline IgG levels before rituximab start** | | | |
| --- | --- | --- | --- |
| *Predictors* | *Estimates* | *Confidence Interval* | *p* |
| **IgG is divided into tertials at baseline (g/L)** |  |  |  |
| IgG lowest tertile | 8,42 | 8.33 – 8.51 | **<0.001** |
| Δ IgG tertile 2 | +2,08 | 1.95 – 2.20 | **<0.001** |
| Δ IgG tertile 3 | +4,44 | 4.28 – 4.60 | **<0.001** |
| **Rate of immunoglobulin change of lowest tertile at baseline, per year (g/L/year)** | -0,15 | -0.20 – -0.11 | **<0.001** |
| ***Additional rate of IgG change compared with the lowest tertile at baseline*** |  |  |  |
| Patients with a baseline IgG at tertile 2 | -0,10 | -0.17 – -0.03 | **<0.001** |
| Patients with a baseline IgG at tertile 3 | -0,23 | -0.31 – -0.15 | **<0.001** |
|  |  |  |  |
| N _ID_ | 1903 |  |  |
| Observations | 9862 |  |  |

Abbreviations: IgG – Immunoglobulin G, g/L – grams per liter, NID – Number of Individuals, Δ – Change in. Baseline IgG levels were categorized into tertials for this analysis, with the lowest tertile serving as the reference category. The Δ IgG represents the average difference in baseline IgG levels for the second and third tertiles compared to the lowest tertile. The rate of immunoglobulin change is reported as an annual decline, calculated from longitudinal data using Generalized Estimating Equations to account for within-subject correlation over time. The additional rate of IgG change compared with the lowest tertile indicates the difference in the rate of decline between the tertiles. Estimates and 95% confidence intervals reflect the magnitude and precision of the IgG change, with p-values indicating the level of statistical significance for each predictor. A p-value of <0.05 was considered statistically significant. These analyses help to elucidate the dependency of IgG decline on baseline IgG levels prior to rituximab initiation.

Supplemental Table S1B

| **Supplemental Table 1B. IgM decline is dependent on baseline IgM levels before rituximab start** | | | |
| --- | --- | --- | --- |
| *Predictors* | *Estimates* | *Confidence Interval* | *p* |
| ***IgM is divided into tertials at baseline (g/L)*** |  |  |  |
| IgM lowest tertile | 0,5 | 0.49 – 0.52 | **<0.001** |
| Δ IgM tertile 2 | 0,43 | 0.41 – 0.45 | **<0.001** |
| Δ IgM tertile 3 | 1,23 | 1.18 – 1.28 | **<0.001** |
| **Rate of immunoglobulin change of lowest tertile at baseline, per year (g/L/year)** | -0,08 | -0.10 – -0.06 | **<0.001** |
| ***Additional rate of IgM change compared with the lowest tertile at baseline*** |  |  |  |
| Patients with a baseline IgM at tertile 2 | -0,13 | -0.17 – -0.10 | **<0.001** |
| Patients with a baseline IgM at tertile 3 | -0,28 | -0.33 – -0.22 | **<0.001** |
| **Squared “Rate of immunoglobulin change of lowest tertile at baseline” (g/L/year)** | 0,01 | 0.01 – 0.01 | **<0.001** |
| ***Additional square rate of IgM change compared with the lowest tertile at baseline***  Squared “patients with a baseline IgM at tertile 2” | 0,02 | 0.01 – 0.02 | **<0.001** |
| Squared “patients with a baseline IgM at tertile 3” | 0,03 | 0.02 – 0.04 | **<0.001** |
|  |  |  |  |
| N _ID_ | 1584 |  |  |
| Observations | 6678 |  |  |

This table builds upon the analyses presented in Supplemental Table 1A, extending the investigation to IgM levels. Baseline IgM is similarly divided into tertiles, and the estimated rates of decline are calculated per annum. The lowest tertile serves as the reference for comparison, and the Δ symbol denotes the mean difference in baseline IgM levels for the middle and upper tertiles relative to the lowest. A squared term is included to adjust for non-linearity in the rate of decline across tertiles.

Supplemental Table S2

| **Supplemental Table 2. Linear regression analysis of age, sex, and prior DMT use on baseline IgG levels in a cohort of before start of rituximab treatment in MS patients** | | | |
| --- | --- | --- | --- |
| *Predictors* | *Estimates* | *Confidence Interval* | *p* |
| **Intercept** **(g/L)** | 10.81 | 10.57 – 11.04 | **<0.001** |
| **Age, quantiles (g/L)** |  |  |  |
| 2nd quantile^b^ | 0.17 | -0.11 – 0.45 | 0.24 |
| 3rd quantile^b^ | -0.00 | -0.29 – 0.29 | 0.99 |
| 4th quantile^b^ | -0.57 | -0.85 – -0.28 | **<0.001** |
| **Sex (g/L)** |  |  |  |
| Male^a^ | 0.17 | -0.41 – 0.08 | 0.18 |
| **Previous DMT (g/L)** |  |  |  |
| Fingolimod ^c^ | -1.13 | -1.52, -0.73 | **<0.001** |
| Natalizumab ^c^ | -0.86 | -1.15 – -0.58 | **<0.001** |
| Teriflunomide ^c^ | -0.88 | -1.59 – -0.18 | **0.014** |
| Other ^c^ | -0.24 | -0.87 – 0.39 | 0.452 |
| Dimethyl fumarate ^c^ | -0.19 | -0.53 – 0.15 | 0.276 |
| Glatiramer acetate ^c^ | 0.31 | -0.24 – 0.86 | 0.268 |
| Interferon β ^c^ | 0.51 | 0.22 – 0.80 | **<0.001** |
| **N _ID_** | 1903 |  |  |
| **R2** | 0.061 |  |  |

Abbreviations: 'IgG' denotes Immunoglobulin G; 'g/L' represents grams per liter; NID – Number of Individuals; 'DMT' refers to disease-modifying therapy, with treatment-naïve patients as the reference group for DMT comparisons. The reference group for sex is females, and for age, it is the lowest quantile. This table utilizes linear regression analysis to explore the impact of age, sex, and prior DMT use on baseline IgG levels before rituximab treatment initiation in multiple sclerosis (MS) patients.

^a^ The comparison group is female pwMS

^b^ The comparison group is the lowest quantile of age at inclusion

^c^ The comparison group is previously untreated pwMS

Supplemental Table S3

|  |  | **Dose of RTX (kilograms)** | | **Interval between doses (days)** | |
| --- | --- | --- | --- | --- | --- |
| **Infusion No** | **Numbers treated** | Mean (SD) | Median [Min, Max] | Mean (SD) | Median [Min, Max] |
| 1 | (N=2743) | 0.804 (0.349) | 1.00 [0.100, 2.00] | 0 (0) | 0 [0, 0] |
| 2 | (N=2591) | 0.618 (0.225) | 0.500 [0.100, 2.00] | 202 (145) | 184 [3.00, 2130] |
| 3 | (N=2315) | 0.605 (0.206) | 0.500 [0.100, 1.00] | 211 (113) | 185 [5.99, 2220] |
| 4 | (N=2092) | 0.596 (0.201) | 0.500 [0.100, 1.00] | 201 (84.5) | 184 [2.00, 1890] |
| 5 | (N=1855) | 0.583 (0.190) | 0.500 [0.100, 1.00] | 208 (81.2) | 185 [14.0, 1070] |
| 6 | (N=1584) | 0.568 (0.180) | 0.500 [0.100, 1.00] | 215 (83.7) | 187 [12.0, 833] |
| 7 | (N=1293) | 0.564 (0.174) | 0.500 [0.0500, 1.00] | 235 (96.3) | 191 [10.0, 954] |
| 8 | (N=946) | 0.550 (0.163) | 0.500 [0.0500, 1.00] | 270 (109) | 231 [10.0, 860] |
| 9 | (N=652) | 0.543 (0.157) | 0.500 [0.100, 1.00] | 273 (107) | 245 [13.0, 775] |
| 10 | (N=416) | 0.523 (0.133) | 0.500 [0.200, 1.00] | 276 (112) | 236 [93.1, 798] |
| 11 | (N=229) | 0.520 (0.132) | 0.500 [0.100, 1.00] | 257 (103) | 205 [14.0, 639] |
| 12 | (N=117) | 0.531 (0.130) | 0.500 [0.300, 1.00] | 239 (77.1) | 212 [90.0, 467] |
| 13 | (N=54) | 0.557 (0.177) | 0.500 [0.300, 1.00] | 242 (94.3) | 195 [91.0, 464] |
| **Overall** | (N=16975) | 0.619 (0.241) | 0.500 [0.0500, 2.00] | 184 (129) | 183 [0, 2220] |

**Supplemental Table 3. Mean and median doses of rituximab (in 1000 grams) and intervals between doses (in days) for the consecutive doses 1-13.** Number of patients receiving each dose is presented as numbers treated. The lowest and highest received individual dose and interval is presented in brackets.
